# Comparison of isoflurane and propofol sedation in critically ill COVID-19 patients—a retrospective chart review

**DOI:** 10.1007/s00540-021-02960-6

**Published:** 2021-06-25

**Authors:** Azzeddine Kermad, Jacques Speltz, Guy Danziger, Thilo Mertke, Robert Bals, Thomas Volk, Philipp M. Lepper, Andreas Meiser

**Affiliations:** 1 Department of Anesthesiology, Intensive Care and Pain Medicine, Saarland University Hospital Medical Center, Kirrberger Str. 100, 66421 Homburg, Saarland Germany; 2 Department of Internal Medicine V-Pulmonology, Allergology and Intensive Care Medicine, Saarland University Hospital Medical Center, Homburg, Saarland Germany

**Keywords:** COVID-19, Inhaled sedation, Isoflurane, AnaConDa, ECMO, Critical care

## Abstract

**Purpose:**

In this retrospective study, we compared inhaled sedation with isoflurane to intravenous propofol in invasively ventilated COVID-19 patients with ARDS (Acute Respiratory Distress Syndrome).

**Methods:**

Charts of all 20 patients with COVID-19 ARDS admitted to the ICU of a German University Hospital during the first wave of the pandemic between 22/03/2020 and 21/04/2020 were reviewed. Among screened 333 days, isoflurane was used in 97 days, while in 187 days, propofol was used for 12 h or more. The effect and dose of these two sedatives were compared. Mixed sedation days were excluded.

**Results:**

Patients’ age (median [interquartile range]) was 64 (60–68) years. They were invasively ventilated for 36 [21–50] days. End-tidal isoflurane concentrations were high (0.96 ± 0.41 Vol %); multiple linear regression yielded the ratio (isoflurane infusion rate)/(minute ventilation) as the single best predictor. Infusion rates were decreased under ECMO (3.5 ± 1.4 versus 7.1 ± 3.2 ml∙h^−1^; *p* < 0.001). In five patients, the maximum recommended dose of propofol of 4 mg∙hour^−1^∙kg^−1^ABW was exceeded on several days. On isoflurane compared to propofol days, neuro-muscular blocking agents (NMBAs) were used less frequently (11% versus 21%; *p* < 0.05), as were co-sedatives (7% versus 31%, *p* < 0.001); daily opioid doses were lower (720 [720–960] versus 1080 [720–1620] mg morphine equivalents, *p* < 0.001); and RASS scores indicated deeper levels of sedation (− 4.0 [− 4.0 to − 3.0] versus − 3.0 [− 3.6 to − 2.5]; *p* < 0.01).

**Conclusion:**

Isoflurane provided sufficient sedation with less NMBAs, less polypharmacy and lower opioid doses compared to propofol. High doses of both drugs were needed in severely ill COVID-19 patients.

## Introduction

Coronavirus disease (COVID-19) and its pandemic spread is a big challenge for health care providers. Patients presenting severe forms of COVID-19 associated with acute respiratory failure are in need for invasive mechanical ventilation and sufficient sedation to ensure adequate gas exchange. Few subjective reports from clinical experience state that COVID-19 patients under mechanical ventilation do need considerably higher doses of intravenous sedatives [1, 2] as well as muscle relaxation [3] with neuromuscular blocking agents (NMBA) to achieve a sufficient depth of sedation and avoid patient–ventilator dyssynchrony. Due to the massive simultaneous worldwide occurrence of severe COVID-19 and requirement of intravenous sedatives, an international shortage of drugs including propofol [4–6] as well as an overrun of intensive care unit (ICU) capacities were observed. Inhaled sedation has shown benefits in animals and patients suffering from ARDS (Acute Respiratory Distress Syndrome) [7–9] and a large number of ICUs seeked for an alternative to intravenous sedation and started to use inhaled sedation [10].

The AnaConDa™ (Sedana Medical, Danderyd, Sweden) is a reflection device working passively, enabling the application of isoflurane or sevoflurane when placed between the Y-piece of the ventilator and the endotracheal tube. The active carbon filter inside retains anesthetic gas molecules in the expiratory phase and releases about 80–90% of them back to the patient in the following inspiration [11].

In this retrospective study, we compared inhaled sedation with isoflurane via AnaConDa to intravenous propofol sedation in COVID-19 patients with ARDS according to the Berlin definition [12]. Sedation depth, dosing of opioids, as well as the necessity of polypharmacy and NMBA use were compared between isoflurane and propofol sedation days. We also analyzed the influence of the minute ventilation and of the extracorporeal membrane oxygenation (ECMO) on isoflurane end-tidal concentrations and isoflurane infusion rates. Mean daily consumptions of both sedatives were calculated to estimate the necessary stock supply in case of similar situations in the future.

## Methods

The ethical committee (Ärztekammer des Saarlandes, 16/09/2020, Bu219/20) approved this retrospective chart review and data analysis and waived the need for individual informed consent.

The study was conducted in a 24-bed pulmonary ICU of a German University Hospital. (Saarland University Medical Center, Homburg, Germany). The inclusion period went from 22 March 2020 to 21 April 2020. All adult patients tested positive for SARS-CoV-2 and in need for invasive ventilation admitted to the University Hospital were treated in this ICU and were included. No pediatric patients are treated in this ICU.

Most patients needed deep sedation for prolonged time periods, which in our institution is achieved using isoflurane or propofol. The choice of sedative was at the discretion of the attending physician. The necessary equipment for the use of isoflurane was limited, so that not all patients could be sedated with it at the same time.

Data collection started on the first day of invasive ventilation in our ICU and stopped after extubation/decanulation, after patient transfer to another hospital, when sedation was discontinued, after patient death or at the latest after 30 days, whichever was first. Apart from biometric and general data, we also recorded doses of sedatives, opioid analgesics, administration of neuro-muscular blocking agents (NMBA), scores of the Richmond Agitation–Sedation Scale (RASS), ventilation and ECMO parameters, each averaged on a daily basis from six a.m. until six a.m. the following day.

Each sedation day was categorized depending on the type of sedative applied:

- “ISO” and “PRO” sedation days: when only isoflurane or only propofol were applied for at least 12 h.
- “Other days”: both isoflurane and propofol were combined or only other sedatives like alpha-2 agonists, benzodiazepines or ketamine were applied.

Subsequently, ISO sedation days were compared with PRO sedation days.

### Isoflurane application and ventilator settings

Inhaled sedation with isoflurane was applied using the AnaConDa administration system. This reflection device was placed between the Y-piece of the ventilator hoses and the endotracheal tube or tracheal cannula. Isoflurane was continuously applied using a syringe pump (Perfusor^®^ Space; B. Braun, Melsungen, Germany). End-tidal isoflurane concentrations were measured after each expiration with a gas monitor (Philips IntelliVue M×800, Hamburg, Germany). Inhaled anesthetics were scavenged by connecting the air outlet of the ventilator to a FlurAbsorb™ canister (Sedana Medical, Danderyd, Sweden). Pressure-controlled ventilation (Duo-PAP) with the possibility of intermittent spontaneous and pressure assisted breaths was used as standard ventilation mode (Hamilton C6 Respirator, Hamilton Medical, Bonaduz, Switzerland).

### ECMO therapy

Veno-venous ECMO-therapy was started in patients with a P/F ratio (PaO_2_/FIO_2_; PaO_2_ partial pressure of oxygen in arterial blood; FIO_2_ fraction of inspired oxygen) smaller than 100 mmHg and/or severely compromised decarboxylation despite high peak ventilation pressures above 30 mbar. Heart–lung support systems (Cardiohelp, Getinge AB, Göteborg, Sweden) were used with femoro-jugular cannulation.

### Necessity of neuromuscular blocking agent

In patients with severe ventilator dyssynchrony, NMBAs were administered, if deepening the sedation was not sufficient to improve the clinical situation.

### Polypharmacy

Polypharmacy was defined as the use of a continuous infusion of a co-sedative during at least 1 h a day. Combination of isoflurane or propofol with co-sedatives was necessary in some cases to ensure a sufficient depth of sedation. RASS-Scores were documented at least once per shift. Co-sedatives used were clonidine, dexmedetomidine, ketamine and midazolam applied as continuous intravenous infusions.

### Conversion of opioids in morphine equivalent doses

Sufentanil, our standard opioid for long-term sedation, was used in 95% of the ISO and PRO sedation days with remifentanil and hydromorphone in the remaining 5%. Different opioids were not combined. The infusion rates were left to the discretion of the attending physician. Opioid doses were converted in morphine equivalent doses using previously published conversion factors [13, 14] to enable comparison (sufentanil 1:1000; hydromorphone 1:7; remifentanil 1:200). For remifentanil, the equivalent dose was divided by 60 to account for the considerably shorter half-life.

### Adjusted body weight

The dosage of propofol was related to the adjusted body weight (ABW) (31):

$$ {\text{ABW}}~ = ~{\text{IBW}}~ + ~\left[ {{\text{Cf }}\left( {{\text{TBW}}~{-}~{\text{IBW}}} \right)} \right]\,\,\,; $$

$$ {\text{Cf}}\, = \,{\text{Correction factor for propofol}}\, = \,0.4\,\,\,; $$

$$ {\text{TBW}}\, = \,{\text{Total Body Weight}};{\text{ IBW}}\, = \,{\text{Ideal Body Weight}}\,\,\,{\text{;}} $$

$$ {\text{IBW}}\,{\text{females}}\, = \,45.5\, + \,[0.9~\, \times ~\,\left( {{\text{Height in cm}} - ~154} \right)]\,\,\,; $$

$$ {\text{IBW}}\,{\text{males}}\, = \,50\, + \,[0.9~\, \times \,~\left( {{\text{Height }}\left( {{\text{cm}}} \right) - ~154} \right)]\,\,\,; $$

### Data evaluation and statistical analysis

Data analyses were conducted using SPSS (SPSS Statistics 27, IBM, Armonk, USA). Continuous variables are expressed as mean ± standard deviation (SD) or as median [interquartile range], if not normally distributed. Data were assessed for normality using the Kolmogorov–Smirnov test. The frequencies were compared using a *χ*^2^ test, parametrical data using a *t* test and non-parametrical data using a Mann–Whitney *U* test. Multiple linear regression analysis was used to determine the best predictive parameters for the end-tidal isoflurane concentration.

A statistical significance is accepted at *p* < 0.05.

## Results

**Fig. 1:**
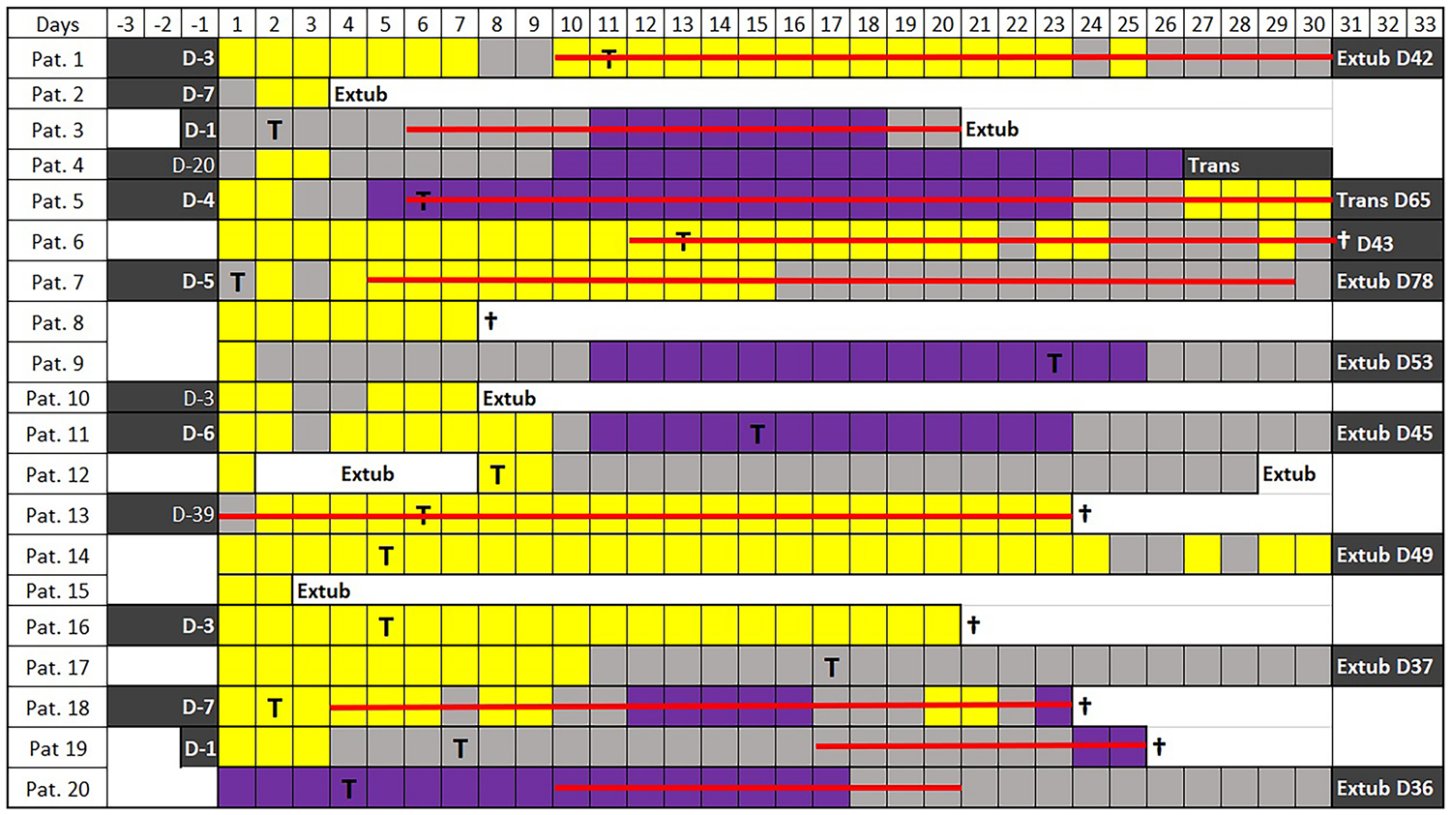
Patient overview. Each box represents 1 day. Yellow box = only propofol was applied for sedation for at least 12 h; purple box = only isoflurane was applied for sedation for at least 12 h; grey box = other days (both, isoflurane and propofol, or other sedatives were used); red line = veno-venous-ECMO; *Pat*. patient; *D *day; *T* day of tracheotomy; *extub*. extubation; *trans* patient transferred to another hospital; † death. Black box = no available data

All 20 patients tested positive for COVID-19 and in need for invasive ventilation in our ICU during the first wave of the pandemic were included. The patients’ biometric and general data are shown in Table 1. Patients were invasively ventilated during 36 [21–50] days. They needed deep sedation, defined by the need to administer isoflurane, or propofol or a combination of both, during 20 [9–24] days. Figure 1 shows the types of sedation days of each patient. In total, 448 sedation days were evaluated. Patients were in need for deep sedation on 333 days. During these, either isoflurane (97 sedation days) or propofol (187 sedation days) or both drugs were used (49 sedation days).

**Table 1:** Patient characteristics and general data

Total number of patients	20
Gender (F: M)	3: 17
Age (years)	64 [60–68]
Height (m)	1.80 [1.75–1.85]
Total body weight (TBW, kg)	96 [85–105]
Adjusted body weight (ABW, kg)	84 [76–88]
BMI (kg/m^2^)	29 [27–32]
BMI > 40 kg/m^2^	3 (15%)
ICU Mortality	7 (35%)
Duration of invasive ventilation (days)	36 [21–50]
Duration of deep sedation (days)	19.5 [9.25–23.5]
Patients receiving VV-ECMO Therapy	9 (45%)
Duration of VV-ECMO (days)	5 [0–22]
Patients receiving isoflurane	8 (40%)
Patients receiving propofol	18 (90%)

Data are median [IQR], or number (frequency)

*VV-ECMO* = veno-venous extracorporeal membrane oxygenation

On ISO sedation days, muscle relaxation and polypharmacy were less frequently necessary and opioid doses were significantly lower compared to PRO days, although RASS-scores were significantly lower on ISO days (Table 2).

**Table 2:** General results

	Isoflurane	Propofol (2%)	*p* value
Number of days	97	187	/
End-tidal isoflurane concentration (Vol. %)	0.96 ± 0.41	/	/
Infusion rate (mL·h^−1^)	5.6 ± 3.1	9.1 ± 4.1	/
Propofol doses (mg∙kg^−1^ABW∙h^−1^)	/	2.2 ± 1.0	/
RASS-Score	− 4.0 [− 3.0 to − 4.0]	− 3.0 [− 3.6 to − 2.5]	< 0.001
Norepinephrine doses (µg·kg^−1^·min^−1)^	0.06 ± 0.05	0.06 ± 0.05	> 0.05
NMBA-use	11 (11%)	(40) 21%	< 0.05
Polypharmacy-use	(7) 7%	(58) 31%	< 0.001
Opioid use (mg·24 h^−1^ morphine equivalent)	720 [720–960]	1080 [720–1620]	< 0.001

Use of neuromuscular blocking agent, polypharmacy, opioid and norepinephrine use in in the ISO and PRO sedation days

On ISO days, isoflurane infusion rates were 5.6 ± 3.1 mL liquid isoflurane per hour (Table 2) and were significantly lower, while patients were on ECMO (3.5 ± 1.4 versus 7.1 ± 3.2 mL·h^−1^, *p* < 0.001).

**Fig. 2:**
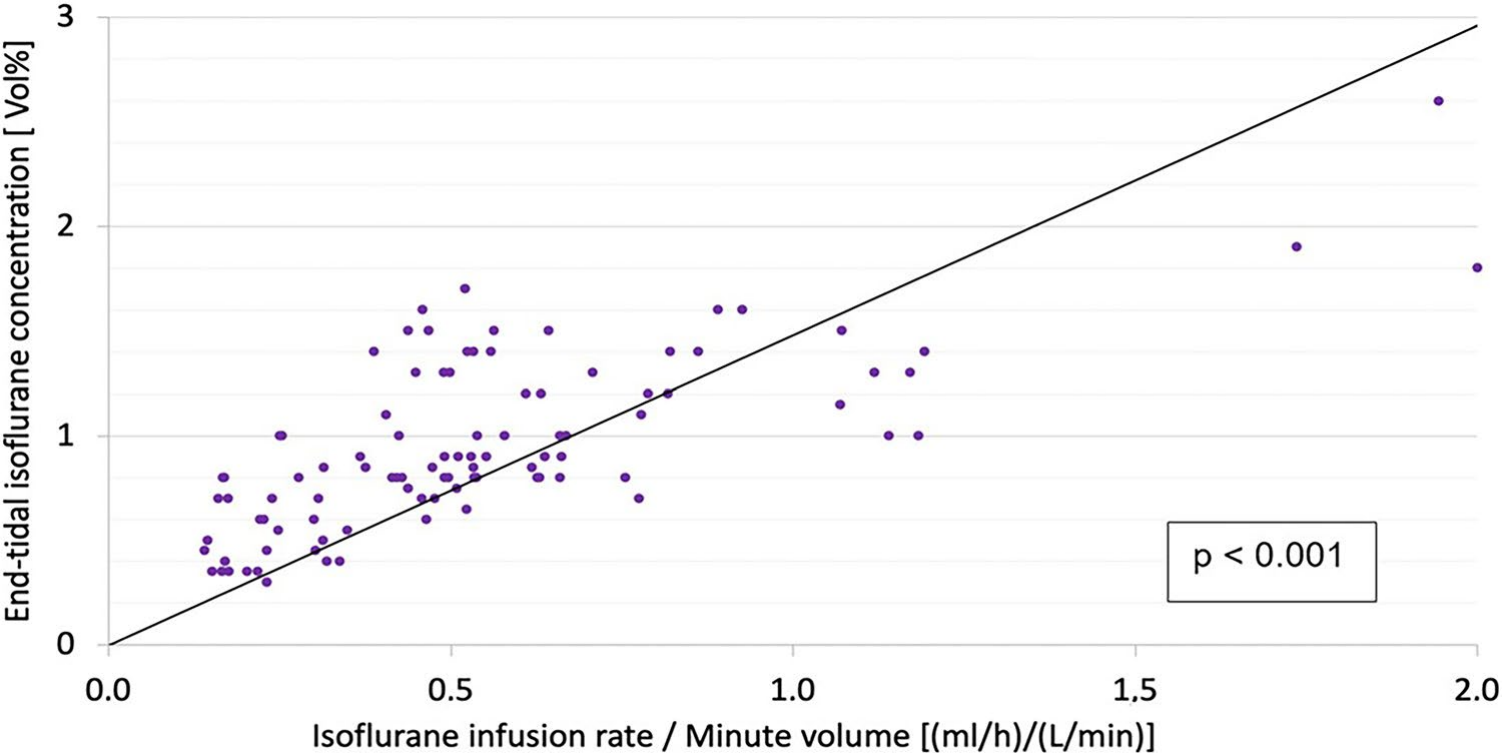
Linear regression model between end-tidal isoflurane concentration and ratio of isoflurane infusion rate/minute ventilation. End-tidal isoflurane concentration correlates poorly with the infusion rate, negatively with the minute ventilation and best with the ratio shown (Spearman’s *R* = 0.7; *p* < 0.001)

Multiple linear regression yielded the ratio infusion rate divided by minute ventilation (Fig. 2) as the single best predictor of the isoflurane end-tidal concentration and a strong correlation was found (Spearman’s; *R* = 0.7; *p* < 0.001). The linear regression analysis established the following equation:

$$ {\text{End}} - {\text{tidal~isoflurane~concentration~}}\left[ {{\text{Vol}}{\text{.~\% }}} \right] = 1.5~ \cdot {\text{Isoflurane~infusion~rate}}~\left[ {{\text{mL}} \cdot {\text{h}}^{{ - 1}} } \right]~/~{\text{Minute~Volume}}~\left[ {{\text{L}} \cdot \min ^{{ - 1}} } \right] . $$

And reciprocally:

$$ {\text{Isoflurane}}\,{\text{infusion}}\,{\text{rate}}\,\,{\text{[mL}} \cdot {\text{h}}^{{ - 1}}] \,{\text{ = }}\,{\text{0.7}}\, \cdot \,{\text{End}}\, - \,{\text{tidal}}\,{\text{isoflurane}}\,{\text{concentration}}\,{\text{[Vol}}{\text{. \% ]}}\, \cdot \,{\text{minute}}\,{\text{volume}}\,{\text{[L}} \cdot {\text{min}}^{{ - 1}}]. $$

On PRO days, propofol doses were 2.2 ± 1.0 mg·kgABW·h^−1^ (Table 2). They did not differ between ECMO and non-ECMO days. The maximum recommended dose of 4.0 mg·kg^−1^·h^−1^ ABW was exceeded in five of our patients on several days.

Vasopressors, almost exclusively norepinephrine, were used more frequently on ISO sedation days. However, norepinephrine doses were low and did not differ between ISO and PRO sedation days (Table 2). Other side effects of isoflurane were not observed.

## Discussion

In this retrospective study, 20 patients with COVID-19 associated ARDS had to be invasively ventilated for long time periods and were in need for deep levels of sedation during a high proportion of that time. The need for deep sedation for prolonged time periods has been reported by others [1–3, 15] and the use of volatile anesthetics has been suggested for COVID-19 patients [9, 10, 16]. In our institution, isoflurane applied with the AnaConDa system or propofol are administered for that purpose.

Dosage of isoflurane may be guided by monitoring its end-tidal concentration. For general anesthesia, concentrations around one minimal alveolar concentration [17] (MAC) are needed, which corresponds to 1.17 (1.03) Vol% isoflurane in patients aged 40 (60) years [18], whereas for sedation in the ICU, lower concentrations corresponding to 0.3 to 0.5 of the MAC have been recommended [19]. In our patients, high concentrations around 1 MAC were needed. Possible explanations include the patients’ relatively young age, previously good health, as well as high respiratory drive [20] and intense inflammatory responses [1]. Medical staff may also have tended for deeper sedation for fear of unplanned extubations associated with a high risk of mortality and viral transmission.

Using a multiple linear regression analysis, we found that neither the isoflurane infusion rate nor the minute volume considered alone can be used to predict the achieved end-tidal isoflurane concentration. In contrast, the ratio (isoflurane infusion rate/minute volume) correlates reliably with that concentration. Thus, knowing the minute volume and the target end-tidal isoflurane concentration, the necessary isoflurane infusion rate can be calculated using the equation established by the linear regression analysis.

In our patients not on ECMO, isoflurane consumption was 170 mL per patient day (drug cost approximately 25 US-$). In other studies, much lower isoflurane infusion rates of 2.1 [17], 3.1 [22] or 4.6 mL·h^−1^ [23] have been described. When ECMO is used, minute ventilation is usually reduced for lung protection. Unlike carbon dioxide, volatile anesthetics do not cross plasma-tight poly-(4-methyl-1-pentene) membranes of modern oxygenators [24, 25]. Thus, during ECMO, isoflurane infusion rates were less than half, and isoflurane consumption was 84 mL per patient day. This information may be useful for managing stock supply of isoflurane.

For propofol, when used for sedation of critically ill patients as opposed to general anesthesia, restrictions are specified in the directions for use [26]: propofol may be used in patients older than 16 years for up to 7 days with a maximal dose of 4 mg·h^−1^ per kg total body weight (TBW). The reason for these restrictions is the occurrence of propofol infusion syndrome (PRIS), a syndrome characterized by metabolic lactic acidosis, cardiac arrythmias, and rhabdomyolysis [27]. PRIS has already been described in a patient suffering from COVID-19 [28]. Furthermore, concomitant use of catecholamines and propofol sedation longer than 48 h increases the incidence of PRIS in intensive care patients [29].

However, in obese patients, applying the specified maximal dose may induce PRIS [30, 31]. This seems important, as many patients with severe COVID-19 are obese [32, 33]. It has been proposed, that propofol should rather be administered based on adjusted (ABW) rather than total body weight (TBW) [34]. For ABW, a drug dependent correction factor is used to account for different volumes of distribution [35].

In our patient collective, the mean BMI corresponds to obesity class 1, leading to a high propofol consumption of 220 mL or 4370 mg per patient day. In fact, in five patients, the propofol dose exceeded 4 mg·kg^−1^·h^−1^ ABW on several days. Despite using relatively high doses of propofol per ABW, RASS-scores evaluated by staff indicated lighter sedation than during isoflurane sedation. Often, sedation with propofol was perceived insufficient and was thus supplemented with co-sedatives or with NMBAs. When using isoflurane, this was done much less frequently. Polypharmacy increases the risk of developing delirium [36]. Also, the opioid dose was significantly lower on isoflurane compared to propofol days. A reduced opioid dose may facilitate spontaneous breathing [37, 38] and promote intestinal motility.

Other possible disadvantages of the use of propofol in COVID-19 patients include the occurrence of hyperlipidemia, which has been associated with an increased risk of mechanical failure of the extracorporeal circuit [15, 39], as well as an increased expression of angiotensin-converting enzyme 2 (ACE-2) in human pulmonary vessels [40]. It is known that the SARS-CoV2 virus uses ACE-2 as a cell entry receptor, and therefore, the use of propofol in these patients has been discouraged [41, 42].

On the other hand, inhaled compared to intravenous sedation has been associated with faster emergence after stop of sedation [43], decreased pulmonary inflammation [44, 45], improved oxygenation in patients with ARDS [8], and even with decreased mortality in a retrospective analysis of long-term ventilated patients [46].

In our study, severe side effects of isoflurane administered during long time periods were not observed. Vasopressors were used more frequently on ISO sedation days, which may be explained by the high concentrations used. Staff working with inhaled sedation needs to be trained to recognize malignant hyperthermia, a life threatening condition. In the case of malignant hyperthermia, application of isoflurane must be stopped immediately and Dantrolene must be administered as soon as possible. Dantrolene must, therefore, be available in all places, where isoflurane sedation is practiced. The genetic predisposition for malignant hyperthermia is very rare (approx. 1:5000–1:100,000)[47] but may vary across countries.

Staff also needs to be trained in the setup of the AnaConDa administration system and the necessary equipment must be acquired. This includes a gas monitor, a syringe pump, and a scavenging device. For passive scavenging, charcoal containers connected to the gas outlet of the ICU ventilator are cheaply available. Daily therapy costs are determined by drug costs (approximately 35 US-$ per 250 ml liquid isoflurane) and by the disposable AnaConDa reflection device (approximately 75 US-$), which is for single patient use and should be exchanged daily. AnaConDa is licensed and commercially available in many countries of the world.

Inhaled sedation with the AnaConDa system has been increasingly used by European ICUs since more than 15 years [21, 23, 43]. The use of isoflurane for ICU sedation is still off-label but is recommended by several national sedation guidelines [49–51]. A phase III registrational study to confirm efficacy and safety of sedation with isoflurane (EudraCT#: 2016-004551-67) has recently been completed [51] and drug approval in the European Union is expected later this year.

### Limitations of this study

In this retrospective chart review, sedation days were classified according to the sedative used and compared with each other. In some cases, both isoflurane and propofol were combined leading to an exclusion of 49 sedation days from a total of 333 days of deep sedation. We did not randomize patients to the sedation regimes under evaluation. The initiation of inhaled sedation depended on the availability of the necessary equipment and on the decision of the treating physician. Inhaled sedation was always used on several consecutive days. Six out of 8 patients received both isoflurane and propofol on different sedation days. It can be assumed that inhaled sedation was chosen in patients difficult to sedate and when intravenous sedation was considered inadequate. Decreased use of NMBAs, additional sedative drugs, and reduced opioid doses on ISO days are, therefore, unlikely to be explained by selection bias.

Sedation days were analyzed as independent variables. However, most differences were highly significant with *p* values well below 0.01, making bias by intra-patient clustering unlikely. Many sedation days were analysed arising from only a small number of patients, which seems justified in this case of pharmacokinetic analyses of dose response relationships.

## Conclusions

In COVID-19 patients, the maximum recommended propofol dose of 4 mg·kg^−1^·h^−1^ ABW may not always be sufficient. In contrast, isoflurane provides sufficiently deep sedation with less polypharmacy, less NMBA use and lower opioid doses.

In this retrospective analysis, the mean daily isoflurane consumption of one COVID-19 patient was 170 mL, reduced to 84 mL when extracorporeal oxygenation was implemented; daily propofol consumption per patient was 4370 mg. For both drugs, these daily doses are higher than for other patients with ARDS. This information may be used for managing stock supply of sedatives during this ongoing pandemic.

## References

[1] Hanidziar D, Bittner EA (2020). Sedation of mechanically ventilated covid-19 patients: challenges and special considerations. Anesth Analg.

[2] Orser BA, Wang DS, Lu WY (2020). Sedating ventilated COVID-19 patients with inhalational anesthetic drugs. EBioMedicine.

[3] Payen JF, Chanques G, Futier E, Velly L, Jaber S, Constantin JM (2020). Sedation for critically ill patients with COVID-19: which specificities? One size does not fit all. Anaesth Crit Care Pain Med.

[4] FDA Drug shortages. Current and resolved drug shortages and discontinuations reported to FDA. https://www.accessdata.fda.gov/scripts/drugshortages/dsp_ActiveIngredientDetails.cfm?AI=PropofolInjectableEmulsion&st=c. Accessed 1 June 2021

[5] Gurascio F. EU scrambles to buy intensive care drugs to tackle covid shortages. Reuters July 8th, 20202. https://www.reuters.com/article/us-health-coronavirus-eu-patients/eu-scrambles-to-buy-intensive-care-drugs-to-tackle-covid-shortages-idUSKBN2492D5. Accessed 1 June 2021

[6] Ammar MA, Sacha GL, Welch SC, Bass SN, Kane-Gill SL, Duggal A, Ammar AA (2020). Sedation, analgesia, and paralysis in covid-19 patients in the setting of drug shortages. J Intensive Care Med.

[7] Ferrando C, Aguilar G, Piqueras L, Soro M, Moreno J, Belda FJ (2013). Sevoflurane, but not propofol, reduces the lung inflammatory response and improves oxygenation in an acute respiratory distress syndrome model: a randomised laboratory study. Eur J Anaesthesiol.

[8] Jabaudon M, Boucher P, Imhoff E, Chabanne R, Faure JS, Roszyk L, Thibault S, Blondonnet R, Vlairefond G, Guérin R, Perbet S, Cayot S, Godet T, Pereire B, Sapin V, Bazin JE, Futier E, Constantin JM. Sevoflurane for sedation in acute respiratory distress syndrome a randomized controlled pilot study. Am J Respir Crit Care Med. 2017;195:792–800. 10.1164/rccm.201604-0686OC. 10.1164/rccm.201604-0686OC 27611637

[9] Jerath A, Ferguson ND, Cuthbertson B (2020). Inhalational volatile-based sedation for COVID-19 pneumonia and ARDS. Intensive Care Med.

[10] Ferrière N, Bodenes L, Bailly P, L’Her E (2020). Shortage of anesthetics: think of inhaled sedation!. J Crit Care.

[11] Kermad A, Speltz J, Daume P, Volk T, Meiser A (2021). Reflection efficiencies of AnaConDa-S and AnaConDa-100 for isoflurane under dry laboratory and simulated clinical conditions: a bench study using a test lung. Expert Rev Med Devices.

[12] The ARDS Definition Task Force. Acute respiratory distress syndrome: the Berlin definition. JAMA. 2012;307:2526–33. 10.1001/jama.2012.5669. 10.1001/jama.2012.5669 22797452

[13] Glass PSA (1995). Remifentanil: a new opioid. J Clin Anesth.

[14] Bhatnagar M, Pruskowski J (2020). Opioid equivalency.

[15] Sherren PB, Ostermann M, Agarwal S, Meadows CIS, Ioannou N, Camporota L (2020). COVID-19-related organ dysfunction and management strategies on the intensive care unit: a narrative review. Br J Anaesth.

[16] Nieuwenhuijs-Moeke GJ, Jainandunsing JS, Struys MMRF (2020). Sevoflurane, a sigh of relief in COVID-19?. Br J Anaesth.

[17] Eger EI, Saidman LJ, Brandstater B (1965). Minimum alveolar anesthetic concentration: a standard of anesthetic potency. Anesthesiology.

[18] Nickalls RWD, Mapleson WW (2003). Age-related iso-MAC charts for isoflurane, sevoflurane and desflurane in man. Br J Anaesth.

[19] Meiser A, Laubenthal H (2005). Inhalational anaesthetics in the ICU: theory and practice of inhalational sedation in the ICU, economics, risk-benefit. Best Pract Res Clin Anaesthesiol.

[20] Gattinoni L, Chiumello D, Caironi P, Busana M, Romitti F, Brazzi L, Camporota L (2020). COVID-19 pneumonia: different respiratory treatments for different phenotypes?. Intensive Care Med.

[21] Sackey PV, Martling CR, Granath F, Radell PJ (2004). Prolonged isoflurane sedation of intensive care unit patients with the anesthetic conserving device. Crit Care Med.

[22] Bomberg H, Meiser F, Zimmer S, Bellgardt M, Volk T, Sessler DI, Groesdonk HV, Meiser A (2018). Halving the volume of AnaConDa: initial clinical experience with a new small-volume anaesthestic reflector in critically ill patients—a quality improvement project. J Clin Monit Comput.

[23] L’Her E, Dy L, Pili R, Prat G, Tonnelier JM, Lefevre M, Renault A, Boles JM (2008). Feasibility and potential cost/benefit of routine isoflurane sedation using an anesthetic-conserving device: a prospective observational study. Respir Care.

[24] Prasser C, Zelenka M, Gruber M, Philipp A, Keyser A, Wiesenack C (2008). Elimination of sevoflurane is reduced in plasma-tight compared to conventional membrane oxygenators. Eur J Anaesthesiol.

[25] Meiser A, Bomberg H, Lepper PM, Trudzinski FC, Volk T, Groesdonk HV (2017). Inhaled sedation in patients with acute respiratory distress syndrome undergoing extracorporeal membrane oxygenation. Anesth Analg.

[26] Propofol-ratiopharm MCT 10 mg / ml Emulsion zur Injektion und Infusion Propofol. Fachinformation Propofol-ratiopharm MCT 10 mg/ml Emulsion zur Injektion und Infusion. Ratiopharm GmbH 2016:1–6. https://www.ratiopharm.de/index.php?eID=dumpFile&t=f&f=73709&g=-1&r=1894%2C1894&token=f727a4d130b2802b5e9ee03d9381563e235f9eed. Accessed 1 June 2021

[27] Hemphill S, McMenamin L, Bellamy MC, Hopkins PM (2019). Propofol infusion syndrome: a structured literature review and analysis of published case reports. Br J Anaesth.

[28] Lucchetta V, Bonvicini D, Ballin A, Tiberio I (2020). Propofol infusion syndrome in severe COVID-19. Br J Anaesth.

[29] Roberts RJ, Barletta JF, Fong JJ, Schumaker G, Kuper PJ, Papadopoulos S (2009). Incidence of propofol-related infusion syndrome in critically ill adults: a prospective, multicenter study. Crit Care.

[30] Ramaiah R, Lollo L, Brannan D, Bhananker S (2011). Propofol infusion syndrome in a super morbidly obese patient (BMI = 75). Int J Crit Illn Inj Sci.

[31] Agarwal A, Greene RA, Shea BS (2018). Rapid onset of propofol infusion syndrome in a super morbidly obese patient. Am J Respir Crit Care Med.

[32] Caussy C, Pattou F, Wallet F, Simon C, Chalopin S, Telliam C, Subtil F, Frobert E, Alligier M, Delaunay D, Vanhems P, Laville M, Jourdain M, Disse E. Prevalence of obesity among adult inpatients with COVID-19 in France. Lancet Diabetes Endocrinol. 2020;8:562–4. 10.1016/S2213-8587(20)30160-1. 10.1016/S2213-8587(20)30160-1 PMC7234780 32437642

[33] Simonnet A, Chetboun M, Poissy J, Raverdy V, Noulette J, Duhamel A, Labreuche J, Mathieu D, Pattou F, Jourdain M (2020). High prevalence of obesity in severe acute respiratory syndrome coronavirus-2 (SARS-CoV-2) requiring invasive mechanical ventilation. Obesity.

[34] Erstad BL, Barletta JF (2020). Drug dosing in the critically ill obese patient—a focus on sedation, analgesia, and delirium. Crit Care.

[35] Macdonald JJ, Moore J, Davey V, Pickering S, Dunne T (2015). The weight debate. J Intensive Care Soc.

[36] Hein C, Forgues A, Piau A, Sommet A, Nourhashémi F, Vellas B, Nourhashémi F (2014). Impact of polypharmacy on occurrence of delirium in elderly emergency patients. J Am Med Dir Assoc.

[37] Heider J, Bansbach J, Kaufmann K, Heinrich S, Loop T, Kalbhenn J (2019). Does volatile sedation with sevoflurane allow spontaneous breathing during prolonged prone positioning in intubated ARDS patients? A retrospective observational feasibility trial. Ann Intensive Care.

[38] Meiser A, Groesdonk HV, Bonnekessel S, Volk T, Bomberg H (2018). Inhalation sedation in subjects with ards undergoing continuous lateral rotational therapy. Respir Care.

[39] Son KH, Lee SI, Choi CH, Park CH (2017). Mechanical failure of extracorporeal membrane oxygenation induced by hypertriglyceridemia. Ann Thorac Surg.

[40] Cao L, Xu L, Huang B, Wu L (2012). Propofol increases angiotensin-converting enzyme 2 expression in human pulmonary artery endothelial cells. Pharmacology.

[41] Sohn JT (2020). Propofol and sedation in patients with coronavirus disease. Am J Emerg Med.

[42] Hirota K, Lambert DG (2020). Propofol and SARS-CoV-2 infection. Br J Anaesth.

[43] Jerath A, Panckhurst J, Parotto M, Lightfoot N, Wasowicz M, Ferguson ND, Steel A, Beattie WS (2017). Safety and efficacy of volatile anesthetic agents compared with standard intravenous midazolam/propofol sedation in ventilated critical care patients: a meta-analysis and systematic review of prospective trials. Anesth Analg.

[44] Voigtsberger S, Lachmann RA, Leutert AC, Schläpfer M, Booy C, Reyes L, Urner M, Schild J, Schimmer RC, Beck-Schimmer B (2009). Sevoflurane ameliorates gas exchange and attenuates lung damage in experimental lipopolysaccharide-induced lung injury. Anesthesiology.

[45] Schläpfer M, Leutert AC, Voigtsberger S, Lachmann RA, Booy C, Beck-Schimmer B (2012). Sevoflurane reduces severity of acute lung injury possibly by impairing formation of alveolar oedema. Clin Exp Immunol.

[46] Bellgardt M, Bomberg H, Herzog-Niescery J, Dasch B, Vogelsang H, Weber TP, Steinfort C, Waldemar U, Wagenpfeil S, Volk T, Meiser A (2016). Survival after long-term isoflurane sedation as opposed to intravenous sedation in critically ill surgical patients. Eur J Anaesthesiol.

[47] Kim DC (2012). Malignant hyperthermia. Korean. J Anesth.

[48] Grounds M, Snelson C, Whitehouse A, Willson J, Tulloch L, Linhartova L, Shah A, Pierson R (2014). Intensive care society review of best practice for analgesia and sedation in the critical care. Intensive Care Soc.

[49] Celis-Rodríguez E, Birchenall C, de la Cal MÁ, Castorena Arellano G, Hernández A, Ceraso D, Díaz Cortés JC, Dueñas Castell C, Jimenez EJ, Meza JC, Muñoz Martínez T, Sosa García JO, Pacheco Tovar C, Pálizas F, Pardo Oviedo JM, Pinilla DI, Raffán-Sanabria F, Raimondi N. Clinical practice guidelines for evidence-based management of sedoanalgesia in critically ill adult patients. Med Intensiva (English Ed). 2013;37:519–74. 10.1016/j.medin.2013.04.001 23773859

[50] DAS-Taskforce. Evidence and consensus based guideline for the management of delirium, analgesia, and sedation in intensive care medicine. Revision 2015 (DAS-guideline 2015)–short version. GMS Ger Med Sci. 2015;13:1–42. 10.3205/000223 PMC4645746 26609286

[51] Meiser A, Volk T, Wallenborn J et al. A randomized controlled trial comparing inhaled isoflurane via the anaesthetic conserving device (Sedaconda; ACD) with propofol for sedation of invasively ventilated ICU patients. Lancet Respir Med. 2021; under review, submitted 21st May 2021. 10.1016/S2213-2600(21)00323-4 34454654

